# Prevalence of Coccidiosis in Free-Range Chicken in Sidi Thabet, Tunisia

**DOI:** 10.1155/2016/7075195

**Published:** 2016-04-24

**Authors:** Khaled Kaboudi, Sajid Umar, Muhammad Tanveer Munir

**Affiliations:** ^1^Department of Poultry Farming and Pathology, National School of Veterinary Medicine, Tunisia; ^2^National Veterinary School of Toulouse, 31300 Toulouse, France; ^3^Department of Pathobiology, PMAS, Arid Agriculture University, Rawalpindi, Pakistan; ^4^Nantes Atlantic National College of Veterinary Medicine, Food Science and Engineering (ONIRIS), Nantes, France

## Abstract

*Background*. Enteric diseases are an important concern to the poultry industry and coccidiosis is imposing a significant economic burden worldwide.* Objectives*. The main goal of the present study was to investigate the prevalence of coccidiosis in free-range chicken in Sidi Thabet, northeast Tunisia.* Methods*. Six hundred and thirty free-range chickens along with fecal samples were collected from 15 flocks in this region and two hundred chickens were found positive for oocysts of* Eimeria *spp. Intestines were dissected and examined for macroscopic lesions. The mucosa of small intestine and the caeca were examined for the presence and identification of parasitic forms using parasitology methods. The mean lesion scores were usually low (<2+) in different intestinal portions of different types of chicken and high scores (>2+) were attributed mainly to the caeca.* Results*. The overall rate of coccidiosis was 31.8%:* E*.* tenella* (61.5%),* E*.* maxima* (12%), and* E*.* acervulina* (1.5%). Mixed* Eimeria* species infection was observed with overall prevalence 26.5%. There was a statistically significant difference (*P* < 0.05) among infection rates, age groups, season, diarrhea, and type of chicken.* Conclusion*. This is the first report of coccidiosis rate in free-range chicken in this region. Further additional studies are needed to develop better preventive measures against coccidiosis in the country.

## 1. Introduction

Enteric diseases are an important concern to the poultry industry because of production losses, increased mortality, reduced welfare of birds and increased risk of contamination of poultry products for human consumption. Avian coccidiosis is an enteric parasitic disease caused by multiple species of the protozoan parasite of the genus* Eimeria*. It is one of the most common and economically most important diseases of poultry worldwide [1, 2]. The disease occurs only after ingestion of sporulated oocysts in susceptible hosts and is characterized by diarrhea, enteritis, emaciation, drooping wings, poor growth, and increased morbidity and mortality. Poor management practices, such as wet litter and high stocking density, can exacerbate the clinical signs [3]. There are seven species of* Eimeria* as causative agents of coccidiosis in chickens, of which* E*.* tenella*,* E*.* maxima*,* E*.* necatrix,* and* E*.* brunetti* are highly pathogenic. Others, like* E*.* acervulina*,* E*.* mitis,* and* E*.* praecox*, are less pathogenic [4–6]. Species of the genus* Eimeria* are predominately host-specific; each species occurs in a single host species or a group of closely related hosts. Most* Eimeria* spp. affect birds between 3 and 18 weeks of age and can cause high mortality in young chicks. Mixed infections are commonly found under field conditions [5]. Coccidiosis is still a major problem worldwide possibly due to poor diagnosis and biosecurity measures. Identification of different species based on morphology of oocysts is very challenging and requires expertise. Differential identification of each species is dependent upon the following characteristics: zone of intestine parasitized, the gross appearance of the lesion, oocyst morphology, minimum sporulation time, minimum prepatent time, schizont size, location of parasite in the host intestinal epithelium, and cross-immunization tests [7].

Poultry sector in Tunisia plays a vital role in income and employment generation as well as being a source of protein. Coccidiosis is a common important disease in commercial and free-range chicken in Tunisia. Mortality and morbidity are usually high and production performances are altered (feed conversion, body weight). Prevention of the disease is based on the hygienic measures, on chemoprevention, and on vaccination. In free-range flocks, coccidiosis is a major cause of mortality of birds because there are no preventive measures. The role of coccidiosis in economic losses of poultry is not clearly recognized in Tunisia. To the best of our knowledge, not a single report has been published on the epidemiological studies on coccidiosis in free-range chicken in Tunisia. This is first report on prevalence rate of coccidiosis in free-range chicken in Tunisia.

## 2. Materials and Methods

### 2.1. Study Area and Sampling

This cross-sectional study was conducted on free-range chickens between September 2010 and August 2015. The total number of birds per flock varied from 30 to 1000 and birds ages were from 4 weeks to 32 weeks. All flocks were situated in and surrounding the village of Sidi Thabet in the Ariana Governorate, Tunisia. The flocks were visited on the basis of abnormal mortality associated with lameness, diarrhea, decreased feed intake, and low body weight. All of the flocks had no history of vaccination against coccidiosis. The chicks and fecal samples were brought to the laboratory for necropsy and parasitology examination, respectively. Information regarding the age, history of diarrhea, and type of chicken and other general information about farms and flocks were taken from farmers.

### 2.2. Sample Examination

The chickens (630) were subjected to routine postmortem examination. All viscera were examined for gross pathological changes at the Avian Pathology Clinical Service of the National Veterinary School of Tunisia. After a general examination of all systems, intestines of each bird were dissected and examined rigorously. Mucosal scrapings of small intestine and the caeca were made and examined microscopically for the presence and identification of oocyst and asexual forms of* Eimeria*. The presence of oocyst in the fecal samples was examined by the flotation method using saturated solution of sugar [3, 4]. The modified McMaster technique was used to quantify the oocyst. For sporulation, positive samples were placed in Petri dishes, conditioned with a solution of 2.5% potassium dichromate at room temperature, and aired daily for up to two days [8]. The* Eimeria* spp. were determined based on morphology of oocysts and sporocysts (shape, color, form index, micropyle, and presence or absence of residual) and time of sporulation [9]. For positive samples, the intensity of the infection was categorized as described by [10] (Table 1).

### 2.3. Statistical Analysis

Statistical analysis was performed by using the software package SPSS version 16.0 for Windows. The differences among variables were evaluated by chi-square test. A *P* value <0.05 was considered statistically significant.

## 3. Results and Discussion

Sidi Thabet is located at the northeast of Tunis city center, around 36°51′45′′N 10°11′44′′E with hot-summer Mediterranean climate, where winters are mild with moderate rainfall and summers are hot and dry. Temperatures in July and August can exceed 40°C (104°F). Winters are mild with temperatures rarely exceeding 20°C (68°F). The Coccidia infection rate in backyard chicken was determined to be 31.8% (Table 1); this rate is low compared to investigations in Iran but higher than studies in Nigeria [11] and Ethiopia [8]. Similarly, Sharma et al. [3] also observed higher prevalence rate (39.58%) of coccidiosis than present study. In previous studies, the infection rate was reported to be 54.3% in Turkey [12], 20.6% and 70.9% in Ethiopia [8, 13], 31.7% and 39.6% in India [3, 14], 36.7% and 52.9% in Nigeria [15], 71.9% in Pakistan [16], 78% in Jordan [17], 88.4% in Argentina [18], and 92% in Romania [19].

Significantly higher (*P* < 0.05) prevalence of clinical coccidiosis was observed in pullets (13%) and broilers (12.5%) than layer (0%). Moreover, Pullet (12.5%) showed significantly higher prevalence of subclinical coccidiosis than layer (3.5%) and broiler (5%) (Table 2). The highest rate of* Eimeria* spp. was determined* by E*.* tenella* (61.5%), followed by* E*.* maxima* (12%) and* E*.* acervulina* (1.5%) (Table 3). These findings are in agreement with Hadipour et al. [20], who reported* E*.* tenella* as the most prevalent species in Iran. However, findings of current study are not in line with the findings of previous studies [21–24]. Our findings are in parallel with reports from Sweden, France, Argentina, and Jordan suggesting that detected species of* Eimeria* are widespread in most countries [17, 18, 25, 26]. Mixed infections with two* Eimeria* spp. were observed in some of the positive farms, with highest frequency of* E. tenella* and* E. maxima* coinfections (25%) followed by coinfections of* E. tenella* and* E. acervulina* (1.5%).

*E*.* acervulina* was only determined in mixed infections with* E*.* tenella*; mixed* Eimeria* infection (26.5%), with highest frequency of the coinfection by* E*.* tenella* and* E*.* maxima* (25%). This may be attributed to the bad management conditions where birds are exposed to a high-risk infection by different pathogens at the same time. In clinical coccidiosis positive samples, the number of oocysts was found to be between 11644 and 38251 per gram (Table 4).

In current investigation, there was a statistical difference among coccidiosis rate, chicken type (*χ* ^2^ = 108.315; df = 2; *P* < 0.05), age groups (*χ* ^2^ = 38.344; df = 2; *P* < 0.05), diarrhea (*χ* ^2^ = 42.884; df = 1; *P* < 0.05), intensity of infection (*χ* ^2^ = 15.510; df = 4; *P* < 0.05), and season (*χ* ^2^ = 22.884; df = 3; *P* < 0.05). Prevalence of infected birds is influenced by the type of chickens (*P* < 0.05) as described in Table 1. It was highest in pullets (17.30%), followed by broilers (12.54%) and layers (1.90%). This finding may be due to the physiognomy of the free-range poultry farming, which was based on the preservation of females, in order to enlarge the flock and increase the egg production. However, males are usually sold in live bird market for consumption as protein source. Similarly, Gharekhani et al. [4] showed higher prevalence of coccidiosis in young chicken (37.5%) of 4–6 wks of age in Iran. Olrija et al. [13] reported higher prevalence of coccidiosis (25.10%) in Bovans breed of chicken in Ethiopia. However, Olanrewaju and Agbor [1] reported higher prevalence of coccidiosis in cockerels (80%) in Nigeria.

The infection was observed all around the year (Table 4) but the prevalence was significantly higher (*P* < 0.05) in the autumn (50.9%) followed by winter (36.9%), spring (36.3%), and summer (3.1%). Our results are in line with Sharma et al. [3] and Khan et al. [16]. This high prevalence in the rainy period in Tunisia could be attributed to increase in humidity and drops in temperature, which is conducive for sporulation of oocysts for easy dispersion and transmission.

Age is one of the most principal factors in coccidiosis. Eimeria spp. can cause infection in all ages of chicken. However, rate of coccidiosis is usually higher in chicken of young ages and the lower rate of coccidiosis of older birds was due to immunity developed by the infection when they were young. In our study, the higher rate of coccidiosis was determined in >10 weeks age groups (17.6%; Table 1); the significant relationship was observed in agreement with other researchers [1, 4, 13, 15, 16, 23]. Sharma et al. [27] reported higher prevalence of coccidiosis in the age group of 31–45 days in broilers. This might be associated with the presence of high number of oocysts in the litter. A possible reason for this may be that during the period between 31 and 45 days of age the birds have not attained immunity against coccidiosis, resulting in the increased incidence of the disease, while the birds of age group 0–15 days were protected by the maternal immunity. Older birds are less susceptible possibly due to enhanced immunity with passage of age. Muazu et al. [15] suggested that all ages of poultry are susceptible to infection but it usually resolves itself around 6–8 weeks of age. It seems that the relationship between age and prevalence rate of coccidiosis is direct due to complete life cycle and the increase of oocysts consumption. Our findings are in accordance with the findings of many authors [28]. Similarly, McDougald and Fitz-Coy [5] reported that coccidiosis is more prevalent in chicken of 3–6 weeks of age.

Diarrhea is a serious sign in clinical coccidiosis [4]. In the present study, 62% of chicks with diarrhea were positive (Table 1). Our results taken with previous investigations are consistent with the idea that the coccidiosis rate correlated with diarrhea. However, our findings do not agree with the findings of Jatau et al. [11] who concluded that local chickens exhibit usually subclinical coccidiosis.

**Figure 1:**
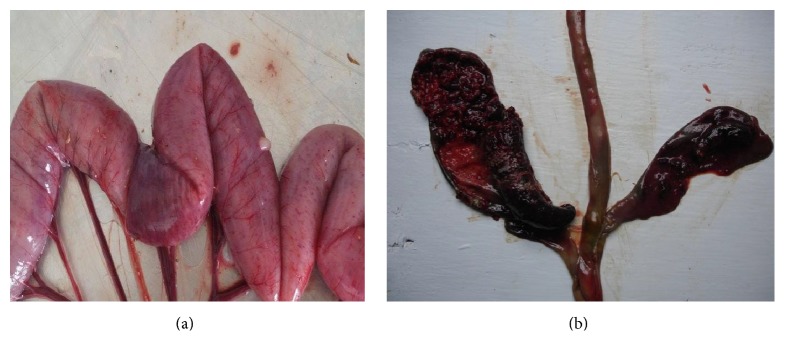
(a) Infected ten-week-old broiler by* E*.* maxima* (note the petechiae through the wall of the middle intestine). (b) Cecal coccidiosis in eight-week-old pullet infected by* E*.* tenella*. The ceca are distended and the wall is thickened. The erosion of the mucosa results in the accumulation of huge amount of fibrin-necrotic material noted at the opening of the cecum.

Necropsy investigation in clinical coccidiosis showed dehydrated and pale carcasses (possibly due to anemia) along with ballooning and hemorrhages in intestines and caeca (Figure 1(a)). Moreover, clotted blood and fibrino-caseous materials in the intestinal lumen were observed in the caeca of broilers and pullets (Figure 1(b)). However, no gross lesions were observed in all free-range chickens with subclinical infection. However, subclinical coccidiosis has been reported to have negative effect on the performance of infected birds [29, 30]. Examination of intestines revealed that high gross lesions were attributed essentially to the caeca followed by duodenum, jejunum/ileum, and colon/rectum segments in broilers and pullets. However, gross lesion scores were significantly higher in jejunum/ileum than duodenum in layers (Table 5). Similar findings have been reported previously by Gari et al. [8] and Raman et al. [31]. Moreover, coccidiosis is a major predisposing factor in the occurrence of necrotic enteritis. Epithelium damage caused by* Eimeria* species allows* Clostridium perfringens* to replicate rapidly and produce toxin, probably because leakage of proteins-rich fluids into the lumen of the gut is favorable to* Clostridium perfringens* proliferation and toxin production [4].

## 4. Conclusion

In conclusion, our findings showed that three pathogenic* Eimeria* species of chicken occurred in free-range poultry in Sidi Thabet, Tunisia with* E*.* tenella* and* E*.* maxima* being the most abundant. Infection occurred mainly in the wet season with high frequency in young chickens. Layer had subclinical coccidiosis. This may pose economic implication to backyard poultry farming due to poor performance of the affected birds that most often looked apparently healthy. This is the first report of coccidiosis rate in free-range poultry in Sidi Thabet, Tunisia. Coccidiosis may be an important factor in the economic losses of the free-range poultry in this region. Therefore, proper control measures must be taken in the form of strict biosecurity, disinfection, vaccination, and good use of anticoccidial drugs. In addition to this, further investigations and design appropriate control strategies in improving management of farms are necessary and strongly recommended.

## Figures and Tables

**Table 1:** Prevalence rate of coccidiosis in free-range chicken in Sidi Thabet, Tunisia.

	Age groups			Birds type			History of diarrhea
	<10 wks	10–20 wks	<20 wks	Broiler	Pullet	Layer	
Number of birds examined (%)	210 (33.33)	210 (33.33)	210 (33.33)	210 (33.33)	210 (33.33)	210 (33.33)	200 (31.7)

Number of positive samples (%)	37 (17.6)	26 (12.38)	4 (1.9)	26 (12.54)	37 (17.30)	4 (1.90)	124 (62)

Number of negative samples (%)	173 (82.3)	184 (87.6)	206 (98.09)	184 (87.6)	173 (82.3)	206 (98.09)	76 (38)

**Table 2:** Prevalence of subclinical and clinical coccidiosis in free-range chicken in Sidi Thabet, Tunisia.

Infection grade	Broiler		Pullet		Layer	
	Number of positive samples	% positive	Number of positive samples	% positive	Number of positive samples	% positive
Subclinical coccidiosis	10	5	25	12.5	7	3.5
Clinical coccidiosis	25	12.5	26	13	0	0

**Table 3:** Prevalence rate of different *Eimeria *species in free-range chicken in Sidi Thabet, Tunisia.

Bird type	Broilers		Pullet		Layer		Prevalence
Eimeria spp.	Number of positive samples	% positive	Number of positive samples	% positive	Number of positive samples	% positive	%
*E. tenella*	50	25	71	35.5	2	1	61.5
*E. maxima*	11	5.5	7	3.5	6	3	12
*E. tenella* + *E. maxima*	17	8.5	29	14.5	4	2	25
*E. tenella* + *E. acervulina*	1	0.5	2	1	0	0	1.5

**Table 4:** Seasonwise prevalence of coccidiosis in free-range chicken in Sidi Thabet, Tunisia.

Season	Number of samples examined	Number of positive samples	Prevalence (%)	OPG (Mean ± SE)
Autumn (September, October, and November)	157	80	50.9	38251.33 ± 1488.2
Winter (December, January, and February)	157	58	36.9	18241.68 ± 756.99
Spring (March, April, and May)	157	57	36.3	15761.27 ± 1245.22
Summer (June, July, and August)	157	5	3.1	11644.23 ± 1987.3

OPG: oocyst per gram.

**Table 5:** Gross lesions attributed to different intestinal segments of infected birds in free-range chicken in Sidi Thabet, Tunisia.

Intestine	Broiler (<10 wks)		Pullet (10–20 wks)		Layer (>20 wks)	
	Number of birds examined	Number of positive samples (%)	Number of birds examined	Number of positive samples (%)	Number of birds examined	Number of positive samples (%)
Duodenum	210	58 (27.6)	210	63 (30)	210	41 (19.5)
Jejunum/ileum	210	30 (14.2)	210	44 (20.9)	210	78 (37.1)
Colon/rectum	210	8 (3.8)	210	11 (5.2)	210	9 (4.2)
Ceca	210	102 (48.5)	210	92 (43.8)	210	82 (39.04)
